# Vertebral fractures among breast cancer survivors in China: a cross-sectional study of prevalence and health services gaps

**DOI:** 10.1186/s12885-018-4014-5

**Published:** 2018-01-30

**Authors:** Evelyn Hsieh, Qin Wang, Renzhi Zhang, Xin Niu, Weibo Xia, Liana Fraenkel, Karl L. Insogna, Jing Li, Jennifer S. Smith, Chunwu Zhou, You-lin Qiao, Pin Zhang

**Affiliations:** 1 Section of Rheumatology, Yale School of Medicine, New Haven, CT USA; 2 Department of Ultrasound, National Cancer Center/Cancer Hospital, Chinese Academy of Medical Sciences & Peking Union Medical College, Beijing, China; 3 Department of Diagnostic Radiology, National Cancer Center/Cancer Hospital, Chinese Academy of Medical Sciences & Peking Union Medical College, Beijing, China; 4 Department of Cancer Epidemiology, National Cancer Center/Cancer Hospital, Chinese Academy of Medical Sciences & Peking Union Medical College, Beijing, China; 5 Department of Endocrinology, Key Laboratory of Endocrinology, Peking Union Medical College Hospital, Beijing, China; 6 Section of Endocrinology, Yale School of Medicine, New Haven, CT USA; 7 Department of Epidemiology, UNC Gillings School of Global Public Health, Chapel Hill, NC USA; 8 Department of Medical Oncology, National Cancer Center/Cancer Hospital, Chinese Academy of Medical Sciences & Peking Union Medical College, Beijing, China

**Keywords:** Breast cancer, Cancer treatment-induced bone loss, Vertebral fracture, China

## Abstract

**Background:**

Breast cancer survivors are at high risk for fracture due to cancer treatment-induced bone loss, however, data is scarce regarding the scope of this problem from an epidemiologic and health services perspective among Chinese women with breast cancer.

**Methods:**

We designed a cross-sectional study comparing prevalence of vertebral fractures among age- and BMI-matched women from two cohorts. Women in the *Breast Cancer Survivors* cohort were enrolled from a large cancer hospital in Beijing. Eligibility criteria included age 50–70 years, initiation of treatment for breast cancer at least 5 years prior to enrollment, and no history of metabolic bone disease or bone metastases. Data collected included sociodemographic characteristics; fracture-related risk factors, screening and preventive measures; breast cancer history; and thoracolumbar x-ray. The matched comparator group was selected from participants enrolled in the *Peking Vertebral Fracture Study*, an independent cohort of healthy community-dwelling postmenopausal women from Beijing.

**Results:**

Two hundred breast cancer survivors were enrolled (mean age 57.5 ± 4.9 years), and compared with 200 matched healthy women. Twenty-two (11%) vertebral fractures were identified among breast cancer survivors compared with 7 (3.5%) vertebral fractures in the comparison group, yielding an adjusted odds ratio for vertebral fracture of 4.16 (95%CI 1.69–10.21, *p* < 0.01). The majority had early stage (85.3%) and estrogen and/or progesterone receptor positive (84.6%) breast cancer. Approximately half of breast cancer survivors reported taking calcium supplements, 6.1% reported taking vitamin D supplements, and only 27% reported having a bone density scan since being diagnosed with breast cancer.

**Conclusions:**

Despite a four-fold increased odds of prevalent vertebral fracture among Chinese breast cancer survivors in our study, rates of screening for osteoporosis and fracture risk were low reflecting a lack of standardization of care regarding cancer-treatment induced bone loss.

## Background

Breast cancer incidence worldwide has risen by 20% and mortality rates by 14% since 2008, with the bulk of this increase sustained by women in low- and middle-income countries (LMICs) due to increasing life expectancy, urbanization, and adoption of Western lifestyles [1]. Although incidence rates have traditionally been low in Asia compared with the U.S. or Europe, due to the sheer population of many Asian countries, the absolute numbers of women with breast cancer in this region has risen dramatically [2].

Several studies have shown that women with breast cancer are at increased risk for osteoporosis and fracture [3–5]. This is largely attributable to the negative impact of breast cancer treatments on skeletal health, which occurs through decreased estrogen exposure [6, 7]. The majority of such studies have been carried out in the U.S. and Europe, where most women are postmenopausal at the time of diagnosis and approximately 75% of patients have hormone-receptor positive disease [8]. By contrast, a nation-wide epidemiologic study found that the average age at breast cancer diagnosis in China is approximately 10 years earlier than in the West, and only 57.4% of women with breast cancer had hormone-receptor positive disease [9].

Because of these differences in epidemiology, risk factors for fracture may be significantly different among Chinese breast cancer survivors, and cannot simply be extrapolated from studies in U.S. and European populations. Furthermore, in China, as in many other LMICs, the infrastructure to monitor and manage osteoporosis and fracture is severely limited, with scarce access to dual-energy x-ray absorptiometry (DXA)—the gold-standard for bone mineral density assessment—outside of large tertiary care centers in major cities [10].

In order to quantify the scope of this problem and identify potential health services gaps, we designed a cross-sectional study to measure the prevalence of vertebral fractures among a cohort of breast cancer survivors in Beijing, and compared our data with fracture prevalence among community-dwelling healthy women in Beijing selected from a pre-existing cohort called the Peking Vertebral Fracture (PK-VF) Study, and hypothesized that vertebral fracture prevalence would be significantly higher among our cohort of breast cancer survivors.

## Methods

### Study design

We conducted a cross-sectional study comparing vertebral fracture rates and risk factors for fracture among two cohorts in Beijing, China: breast cancer survivors currently receiving care at a single large cancer center, and an age- and body mass index (BMI)-matched group of community-dwelling healthy women recruited as part of a pre-existing epidemiological study of vertebral fracture prevalence in Beijing. This study was reviewed and approved by the institutional review board of the Cancer Hospital, Chinese Academy of Medical Sciences and the human investigations committee of Yale School of Medicine prior to initiation.

### Sample

#### Breast cancer survivors cohort

All breast cancer survivors receiving care at the Cancer Hospital, Chinese Academy of Medical Sciences (CHCAMS) from April 1, 2013 – August 31, 2014 were eligible for this study if they met the following criteria: 1) age 50–70 years, 2) initiated breast cancer treatment at least 5 years prior to enrollment. Exclusion criteria included: 1) initiated breast cancer therapy within last 5 years, 2) history of bone metastases, 2) osteoporosis or osteoporosis therapy prior to breast cancer diagnosis, 3) metabolic or inherited bone disease, 4) corticosteroid or anticonvulsive therapy for > 6 months or within the last 12 months, 5) conditions leading to secondary osteoporosis (rheumatoid arthritis/connective tissue disease, chronic liver or kidney disease, malabsorption, inflammatory bowel disease, poorly controlled hyperthyroidism). Initially women were recruited by screening the CHCAMS medical database for women diagnosed with breast cancer at CHCAMS prior to January 1, 2008. Potentially eligible women were contacted via telephone and invited to participate in the study. However due to the low success rate of this method during the first month, we switched to a physician referral recruitment approach where all patients presenting for follow up in the breast cancer clinic who met eligibility criteria were referred to the study physician. The study physician met with each potential participant, confirmed eligibility, explained the study purpose, procedures and risks and benefits, and obtained written informed consent.

#### PK-VF cohort

Details regarding the recruitment of the PK-VF Study have been described previously [11]. This study recruited a total of 1724 postmenopausal community-dwelling Chinese women in 2008 from seven districts of Beijing, aged 47–108 years. Investigators collected detailed data regarding sociodemographic and clinical characteristics, osteoporosis and fracture-related history and risk factors, thoraco-lumbar x-rays, and serologic samples. For the purposes of this study, we randomly selected an age- and BMI-matched sample of women from the PK-VF cohort.

### Measures

#### Breast cancer survivors cohort

Sources of data for each participant included a self-administered study questionnaire, a medical chart review form completed by the study physician, thoracolumbar x-ray, and fasting serum sample. The study questionnaire consisted of 19 questions that addressed sociodemographic characteristics (age, education level, smoking history, and current alcohol use), history of and risk factors for fracture (height, weight, history of a fall within the last year, parental history of fracture, personal history of fracture, bone mineral density testing or diagnosis of low bone mineral density since breast cancer diagnosis, calcium or vitamin D supplement use), and reproductive health history (age at menarche, parity, age at menopause if applicable, and history of hormone replacement therapy use). Data collected from the medical chart included date of breast cancer diagnosis, stage at diagnosis, pathologic diagnosis, estrogen- and progesterone-receptor status, and HER-2 receptor status. Initial breast cancer treatment history was recorded including type of surgery, and use of radiation therapy, chemotherapy, and/or endocrine therapy [i.e. selective estrogen-receptor modulators (SERMs), aromatase inhibitors (AIs), gonadotropin releasing hormone (GnRH) agonists, ovariectomy]. Data regarding history of recurrence were also obtained.

Thoracic and lumbar lateral spine x-rays were performed to identify prevalent vertebral fractures at the diagnostic imaging department of CHCAMS. For each participant, presence of vertebral fracture was ascertained by two radiologists using the validated *Genant semi-quantitative technique* [12]. In this method, vertebrae T4-L4 are graded on visual inspection and without direct measurement at normal (grade 0), mildly deformed (grade 1, approximately 20–25% reduction in anterior, middle, and/or posterior height and a reduction in area of 10–20%), moderately deformed (grade 2, approximately 25–40 reduction in any height and a reduction in area 20–40%), and severely deformed (grade 3, approximately 40% reduction in any height and area).

Fasting serum samples were collected from each participant and stored at -80 °C until batch testing at the end of the study period. We tested 25-hydroxy vitamin D (25OHD) levels, and the bone formation marker pro-collagen type 1 N propeptide (P1NP), and the bone resorption marker serum β-c-terminal telopeptide of type 1 collagen (CTx) to assess bone metabolism. All biomarkers were assayed at the Di’an laboratory in Beijing, China using an automated Roche electrochemiluminescence immunoassay (cobas e 601, Roche Diagnostics, Basel, Switzerland).

#### PK-VF cohort

For each participant, data were extracted from the PK-VF Study database regarding participant age, BMI, education level, parity, menstrual history, smoking, alcohol use, history of bilateral ovariectomy, parental history of fracture, personal history of fracture, and calcium supplement use. As part of the PK-VF Study, prevalent vertebral fractures were also ascertained using lateral thoracolumbar x-ray, and evaluated by two experienced radiologists using the Genant semi-quantitative method described above. Serum biomarkers, CTx, P1NP, and 25OHD, were batch tested at the central laboratory of the Department of Endocrinology, Peking Union Medical College Hospital, by an automated Roche electrochemiluminescence immunoassay (Modular Analytics E170; Roche Diagnostics, Basel Switzerland) [11].

### Data analysis

All statistical analyses were performed using Stata Intercooled 13 (StataCorp, College Station, TX). Descriptive statistics were used to report the sociodemographic characteristics, frequency of fracture, and fracture-related risk factors in the two cohorts. Categorical variables were compared using χ^2^ or Fisher’s exact test, and continuous variables were compared using the t-test. Univariate logistic regression was further used to calculate the odds of vertebral fracture based upon breast cancer status, age, BMI, level of education, parity, personal history of fracture, calcium supplement use, and 25OHD level. We fit the multivariable model using backward elimination beginning with all variables that were significant (*p*-value < 0.10) in the univariate analyses (breast cancer survivor status, age, parity, calcium supplement use) [13]. To obtain a parsimonious model, we removed non-significant variables (*p* > 0.05) one at a time beginning with the least significant; in each step, remaining parameter estimates remained largely unchanged (< 20%). Among the cohort of breast cancer survivors, we further performed subgroup analyses based upon fracture status using χ^2^, Fisher’s exact test, and the t-test as appropriate.

## Results

### Sociodemographic, reproductive and fracture-associated characteristics

In total, 200 survivors of breast cancer were enrolled and 200 matched healthy women were selected from the PK-VF cohort. The mean age of both cohorts was 57.5 ± 4.9 years and over half of women had a BMI above 24 kg/m^2^ (Table 1). Women in the breast cancer cohort were more highly educated and smoking and alcohol use were rare among both cohorts. Fewer women with breast cancer self-reported a personal history of fracture (10.5 v. 21%, *p* = 0.004). Among breast cancer survivors the most commonly reported site of fracture was the wrist (8/21, 38.1%). One patient reported a history of hip fracture and only one self-reported a history of vertebral fracture. Other reported fracture sites included the ankle, lower leg, foot, finger, coccyx, knee, toes, and ribs. Thirty-three percent (6/18) of fractures were reported as low-trauma fractures. More breast cancer survivors reported using calcium supplements (49 v. 36.9%, *p* = 0.015). Only 12/200 (6%) of women with breast cancer reported using vitamin D supplements.

**Table 1:** Sociodemographic, Reproductive, and Fracture-Associated Characteristics of BCS and PK-VF Study Cohorts

Variable	BCS Cohort	PK-VF Cohort
Age *years*	57.5 ± 4.9	57.5 ± 4.9
BMI *kg/m*^*2*^	24.8 ± 3.7	24.7 ± 3.2
Education ≥ High School	156/200 (78)^c^	91/197 (46.1)
Smoking, Ever	6/200 (3)	5/200 (2.5)
Current Alcohol Use	11/200 (5.5)	8/200 (4.0)
Menarche *years*	14.4 ± 2.0^b^	15.1 ± 2.4
Menopause *years*	49.4 ± 4.0	49.6 ± 3.5
Parity		
0	2/200 (1%)	2/190 (1.6)
1	137/200 (68.5)^b^	102/190 (53.7)
≥ 2	61/200 (30.5)^a^	85/190 (44.7)
Fall in Past Year	31/198 (15.7)	–
Parental Fracture History	20/188 (10.6)	31/200 (15.5)
Personal Fracture History	21/200 (10.5)^b^	42/200 (21)
Calcium Supplement Use	96/196 (49.0)^a^	73/198 (36.9)
Vitamin D Supplement Use	12/200 (6.0%)	–
CTx *ng/mL*	0.458 ± 0.211	0.441 ± 0.199
P1NP *ng/mL*	61.1 ± 30.4	56.3 ± 24.6
25OHD *ng/mL*	20.3 ± 7.8^c^	13.3 ± 5.7

Values for continuous variables are reported as *mean ± SD* and for categorical values as *n/N(%)*

*PK-VF* Peking Vertebral Fracture study, *BCS* breast cancer survivors, *CTx* serum β-c-terminal telopeptide of type 1 collagen, *P1NP* pro-collagen type 1 N propeptide, *25OHD* 25-hydroxy vitamin D, *kg* kilograms, *m* meters, *ng* nanograms, *mL* milliliters

^a^ < .05

^b^ < .01

^c^ < .001

### Vitamin D status and bone turnover markers

Mean levels of 25OHD were significantly higher among the breast cancer survivors compared with women in the PK-VF Study (20.3 ± 7.8 v. 13.3 ± 5.7, *p* < 0.001) (Table 1). Even so, 113/196 (58.2%) of women with breast cancer met criteria for 25OHD deficiency (< 20 ng/mL), and 61/196 (35.2%) met criteria for 25OHD insufficiency (20-29 ng/mL). By contrast, no significant differences in mean levels of bone turnover makers were noted between the two groups. The correlation coefficient between CTx and P1NP was 0.72 (*p* < 0.001) among breast cancer survivors, and 0.78 (p < 0.001) among the PK-VF cohort demonstrating appropriate coupling of bone formation and resorption in both groups.

### Vertebral fractures

Table 2 demonstrates the total number of women in the breast cancer survivors cohort (22/200, 11%) and PK-VF cohort (7/200, 3.5%) with prevalent vertebral fractures. Several women had more than one fracture, and the total number of vertebral fractures identified by thoracolumbar x-ray was also significantly greater among breast cancer survivors (47 v. 9 fractures, p < 0.001). Breast cancer survivors were more likely to have multiple fractures as well as higher grade fractures. The odds of having a vertebral fracture among breast cancer survivors compared with their healthy counterparts was 3.41 (95%CI 1.42–8.17, *p* = 0.006) (Table 3). In the multivariable model, the odds of vertebral fracture among breast cancer survivors was 4.16 (95%CI 1.69–10.21, *p* = 0.002) compared with women in the PK-VF cohort.

**Table 2:** Vertebral Fracture Results for BCS and PK-VF Study Cohorts

Variable	BCS Cohort	PK-VF Cohort
Individuals with VF *n/N(%)*	22/200 (11%)^§^	7/200 (3.5%)
Grade 1^a, b^	13	5
Grade 2	12^§^	2
Grade 3	2	0
VFs per Individual *median(range)*	1(1–10)	1(1–2)
Total Number of VF *N*	47^¥^	9
Grade 1	19*	7
Grade 2	25^¥^	2
Grade 3	3	0

*VF* vertebral fracture, *PK-VF* Peking Vertebral Fracture Study, *BCS* breast cancer survivors

^*^ < .05

^§^ < .01

^¥^ < .001

^a^ Please note that because some individuals have multiple fractures of different grades, the number of individuals with grade 1, grade 2 and grade 3 fractures add up to more than 22

^b^ Vertebral Fractures were classified using the Genant Semi-Quantitative technique. In this method, vertebrae T4-L4 are graded as normal (Grade 0), mildly deformed (Grade 1, approximately 20–25% reduction in anterior, middle, and/or posterior height and a reduction in area of 10–20%), moderately deformed (Grade 2, approximately 25–40 reduction in any height and a reduction in area 20–40%), and severely deformed (Grade 3, approximately 40% reduction in any height and area)^13^

**Table 3:** Logistic Regression Analysis: Odds of Vertebral Fracture among Women in the BCS and PK-VF Study Cohorts, Combined (*N* = 400)

Variable	Univariate Model		Multivariable Model	
	OR	95%CI	OR	95%CI
Breast Cancer Survivor	3.41	1.42–8.17^c^	4.16	1.69–10.21^c^
Age *years*	1.08	1.01–1.17^c^	1.10	1.02–1.20^b^
BMI *kg/m*^*2*^	0.90	0.78–1.03	–	–
Education ≥High School	0.85	0.39–1.83	–	–
Parity	1.49	0.93–2.37^a^	–	–
Age of Menarche *years*	1.08	0.92–1.27	–	–
Personal History of Fracture	0.60	0.18–0.12	–	–
Calcium Supplement Use	0.48	0.21–1.12^a^	0.37	0.15–0.89^b^
25OHD Level *ng/mL*	1.00	0.95–1.05	–	–

Continuous variables: Age, BMI, Parity, Age of Menarche, 25OHD level. Categorical variables: Breast Cancer Survivor (reference: Peking Vertebral Fracture Study participant), Education (reference: ≤middle school), Personal History of Fracture (reference: no history of fracture), Calcium Supplement Use (reference: no supplement use)

*OR* odds ratio, *CI* confidence interval, *BMI* body mass index, *25OHD* 25-hydroxy vitamin D

^a^ < 0.1

^b^ < .05

^c^ < .01

### Characteristics of breast cancer survivors cohort

Table 4 details the characteristics of the cohort of breast cancer survivors based upon fracture status, including breast cancer-related characteristics. The average duration of breast cancer at the time of enrollment was 6.3 ± 1.9 years. Approximately 85% of women were diagnosed at early stage (stage I or II), and a similar proportion had hormone receptor positive disease on pathology and underwent some form of endocrine therapy during the course of treatment, including SERMS [tamoxifen (56/70) and/or toremifene (21/70)], AIs [Anastrozole (55/90), Letrozole (23/90), and/or Exemestane (13/90)] and GnRH agonists [Goserelin (1/2) and Leuprolide (1/2)]. Sixteen women underwent ovariectomy as part of breast cancer therapy. The average length of treatment with SERMS was 50.5 ± 21.4 months, and the average length of treatment with AIs was 58.9 ± 17.9 months. Forty-three percent of women were postmenopausal at the time of breast cancer diagnosis, of whom only 29.6% reported having had a DXA scan since being diagnosed. Of the 90 women who were treated with AI therapy, only 30% reported having had a DXA scan. Approximately 16% of breast cancer survivors reported a fall within the last year, and 49% reported taking a calcium supplement.

**Table 4:** Characteristics of BCS Cohort, Overall and by Fracture Status

Variable	Overall	No Fracture	Fracture
Age *years*	57.5 ± 4.9	57.1 ± 4.8	60.0 ± 5.2^b^
BMI *kg/m*^*2*^	24.8 ± 3.7	24.9 ± 3.7	23.8 ± 3.3
Education ≥ High School	156/200 (78)	143/178 (80.3)	13/22 (59.1)^a^
Menarche *years*	14.4 ± 2.0	14.5 ± 1.9	14.5 ± 2.1
Menopause *years*	49.4 ± 4.0	49.4 ± 4.2	49.2 ± 2.2
Parity			
0	2/200 (1)	2/178 (1.1)	0/22 (0)
1	137/200 (68.5)	126/178 (70.8)	11/22 (50)^a^
≥ 2	61/200 (30.5)	50/178 (28.1)	11/22 (50)^a^
Smoking, Ever	6/200 (3)	6/178 (3.4)	0/22 (0)
Current Alcohol Use	11/200 (5.5)	11/178 (6.2)	0/22 (0)
Fall in Past Year	31/198 (15.7)	28/176 (15.9)	3/22 (13.6)
Parental Fracture History	20/188 (10.6)	18/167 (10.8)	2/21 (9.5)
Personal Fracture History	21/200 (10.5)	19/178 (10.7)	2/22 (9.1)
Calcium Supplement	96/196 (49)	88/174 (50.6)	8/22 (36.4)
DXA Since Diagnosis	54/200 (27)	44/178 (24.7)	10/22 (45.5)^a^
Low BMD Since Diagnosis	24/195 (12.3)	22/173 (12.7)	2/22 (9.1)
CTx *ng/mL*	0.458 ± 0.211	0.457 ± 0.209	0.463 ± 0.227
P1NP *ng/mL*	61.1 ± 30.4	61.3 ± 30.1	58.9 ± 33.2
25OHD *ng/mL*	20.3 ± 7.8	20.5 ± 7.6	19.0 ± 9.7
Postmenopausal at Diagnosis	81/189 (42.9)	67/167 (40.1)	14/22 (63.6)^a^
Duration of Breast Cancer	6.3 ± 1.9	6.4 ± 2.0	6.1 ± 1.6
BrCa Stage at Diagnosis			
I	77/183 (42.1)	69/162 (42.6)	8/21 (38.1)
II	79/183 (43.2)	69/162 (42.6)	10/21 (47.6)
III	27/183 (14.8)	24/162 (14.8)	3/21 (14.3)
Hormone Receptor Status			
ER+/PR+	113/186 (60.1)	100/164 (61)	13/22 (59.1)
ER+/PR-	11/186 (5.9)	10/164 (6.1)	1/22 (4.6)
ER-/PR+	33/186 (17.6)	30/164 (18.3)	3/22 (13.6)
ER-/PR-	29/186 (15.4)	24/164 (14.6)	5/22 (22.7)
HER2 Receptor Positive	38/183 (20.8)	35/161 (21.7)	3/22 (13.6)
Surgery	187/187 (100)	165/165 (100)	22/22 (100)
Radiation Therapy	53/182 (29.1)	45/160 (28.1)	8/22 (36.4)
Chemotherapy	153/185 (82.7)	135/163 (82.8)	18/22 (81.8)
Endocrine Therapy	158/189 (84)	141/167 (84.4)	17/22 (77.3)
SERM	70/188 (37.2)	64/166 (38.6)	6/22 (27.3)
Aromatase Inhibitor	90/189 (47.6)	82/167 (49.1)	8/22 (36.4)
GnRH agonist	2/189 (1.1)	2/167 (1.2)	0/22 (0)
Ovariectomy	16/189 (8.5)	13/167(7.8)	3/22 (13.6)
Recurrence	2/185 (1.1)	2/162 (1.2)	0/22 (0)

Values for continuous variables are reported as *mean ± SD* and for categorical values as *n/N(%)*

*PK-VF* Peking Vertebral Fracture Study, *BCS* breast cancer survivors, *DXA* dual-energy x-ray absorptiometry, *BMD* bone mineral density, *CTx* serum β-c-terminal telopeptide of type 1 collagen, *P1NP* pro-collagen type 1 N propeptide, *25OHD* 25-hydroxy vitamin D, *ER* estrogen receptor, *PR* progesterone receptor, *HER2* human epidermal growth factor receptor 2, *SERM* selective estrogen receptor modulator, *GnRH* gonadotropin releasing hormone

^a^ < .05

^b^ < .01

Stratified analyses based upon fracture status, showed that women with fracture were older and therefore more likely to be postmenopausal at the time of diagnosis (63.6 v. 40.1%, *p* = 0.036), and had a lower level of education (59.1 v. 80.3% with ≥ high school education, *p* = 0.023). Although our sample was not powered to formally evaluate differences in fracture status based upon treatment class, we observed that vertebral fractures were detected among 7/74 (9.4%) of women receiving AIs only, 5/54 (9.3%) receiving SERMs only, 1/16 (6.3%) who had received both an AI and SERM, and in 3/16 (18.8%) women who had been treated with ovariectomy. Of note, all three of the ovariectomized women with fractures were premenopausal at the time of diagnosis and had concurrently been treated with a SERM. 8/54 (14.8%) of women who received radiation treatment had a vertebral fracture, however 7 of those women were also concurrently treated with some form of endocrine therapy.

Levels of 25OHD, bone turnover markers, and treatment patterns did not vary based upon fracture status.

## Discussion

Our study is the first to directly measure rates of fracture among breast cancer survivors in China compared to age- and BMI-matched healthy women. We found that history of breast cancer was associated with a four-fold increased odds of prevalent vertebral fractures. Furthermore, breast cancer survivors with fractures were more likely to have multiple fractures and higher grade fractures compared to their healthy counterparts. Finally, rates of DXA screening for bone disease were low among women with breast cancer, even among those who were postmenopausal at the time of diagnosis or treated with AIs, factors known to increase risk for fracture.

Rates of fractures have traditionally been higher in Europe and the U.S. compared to China [14]. Therefore, it is consistent that absolute prevalence of fractures in both of our cohorts were lower compared with previous studies among women from Caucasian populations. However, recent studies have also shown that rates of fracture are rapidly increasing in China due to urbanization and adoption of Western lifestyles [15]. In our study, the magnitude of the increased odds for fracture seen among breast cancer survivors is roughly on par with prior findings. Kanis et al., found that prevalence of vertebral fractures among women with soft-tissue breast cancer recurrence (without skeletal metastases) was six-fold higher compared with healthy controls or women newly diagnosed with breast cancer [4]. However, their study population was acutely ill and mean age was 2 years older than our cohort, whereas our cohort included predominantly recurrence-free breast cancer survivors.

To our knowledge only one prior study has been published from mainland China examining this issue [16]. This study retrospectively compared 70 postmenopausal women with breast cancer receiving AI therapy, with 52 women receiving tamoxifen, and 89 women who were not treated with endocrine therapy at a single institution. The authors found that women on AIs had an increased incidence of fractures (12.9%) compared with those not treated with endocrine therapy (1.1%, *p* = 0.001). By contrast, incidence of fractures among those treated with tamoxifen did not differ (1.9%, *p* = 0.372) compared with those not treated with endocrine therapy. Unfortunately, this study is significantly limited by the lack of description regarding how osteoporotic fractures were defined and measured, and sparse risk factor data.

Three studies from Taiwan using retrospective data from their National Insurance Research Database have also examined this issue among ethnically Chinese women with breast cancer. Tzeng et al. reported tamoxifen use was associated with reduced risk of osteoporotic fractures, however did not specify whether women in their cohort were pre- or postmenopausal [17]. Chang et al. studied fracture risk in young breast cancer patients and found that those receiving AIs, radiotherapy, or Herceptin were at increased risk for future fracture [18]. Tsa et al. showed that age-specific hazard ratio for fracture was higher for breast cancer patients < 50 years of age, both for traumatic and non-traumatic fractures [19]. However, these studies are all based upon insurance claims data and rely on International Conference for the Ninth Revision of the International Classification of Diseases (ICD-9) codes for diagnosis. Validation of the diagnostic codes was not described therefore further studies are needed to confirm these findings in prospective cohorts. Our data provide a meaningful comparison to these studies given the differences in lifestyle, nutrition, medical care and environmental exposures among women in mainland China compared with Taiwan, and provides direct assessment of fracture associated risk factors not available from insurance claims data.

Previous epidemiologic studies from Asian countries have demonstrated that the peak age of breast cancer diagnosis occurs approximately a decade earlier among Asian women compared with their Western counterparts [20, 21]. The age and menopausal status at diagnosis of our cohort are consistent with data from a nationally representative sample of 4211 Chinese women diagnosed with breast cancer (mean age at diagnosis = 48.7 ± 10.5 years, 62.9% premenopausal). Because the vast majority of studies on this subject have been conducted in Western populations, the long-term impact of breast cancer treatment on bone health among Asian women remains unknown. Furthermore, less is known about the long-term risk for fracture among premenopausal women and guidelines cannot simply be extrapolated from studies for postmenopausal women with breast cancer.

As a population, Chinese women undergoing treatment for breast cancer will become vulnerable to fracture at a younger age relative to their Western counterparts, during a period when comparatively little attention is typically given to fracture risk reduction. Furthermore, even when increased fracture risk is identified, management algorithms are less straightforward for premenopausal women compared with postmenopausal women. In 2012, Hadji et al. published a review of the complexities of cancer treatment-induced bone loss (CTIBL) among premenopausal women, and proposed a framework with which to approach this problem including indications for DXA screening and calcium and vitamin D supplementation [22]. However, in China, as in other rapidly modernizing Asian countries, lack of access to DXA and osteoporosis specialists is common, and presents a barrier to comprehensive fracture risk assessment. In 2013, there were only an estimated 0.0046 DXA machines per 10,000 individuals in China, which is far below the density (0.11 per 10,000 population) recommended by the International Osteoporosis Foundation [10]. The lack of DXA access at even the highest-tier cancer centers, including CHCAMS, also suggests a fundamental lack of recognition and prioritization at the health systems level regarding the long-term impact of CTIBL on health outcomes.

From a practice perspective, our study underscores the importance of awareness of fracture risk associated with breast cancer therapies both on the part of the provider and patient. Although fracture rates were relatively high, due to the asymptomatic nature of most vertebral fractures, the vast majority of women and their providers were not aware of these fractures. While guidelines exist in China for screening and management of CTIBL, it is apparent that gaps remain in terms of uptake of these recommendations in practice. Indeed, among breast cancer survivors with a prevalent vertebral fracture, less than 50% had obtained a DXA scan. While 49% of breast cancer survivors were on calcium supplements, only 9% were taking vitamin D supplements. Given age and postmenopausal status are known risk factors for fractures, it is not surprising that overall, more fractures occurred among women who were postmenopausal at the time of diagnosis. However, it is important to note that 8/108 (7.4%) of breast cancer survivors who were premenopausal at the time of diagnosis were also found to have vertebral fractures, which underscores the excess risk for fractures in this population. While studies have demonstrated bisphosphonates may be effective for mitigating CTIBL, such studies have not been powered to measure impact on fracture rates [23–25].

Our study has several limitations that warrant mention. First, the PK-VF cohort has a few key differences compared with the breast cancer survivors cohort. It was enrolled 5 years earlier than the breast cancer survivors cohort, and carried out by a separate group of investigators and radiologists. However, the principal investigator of the PK-VF was a collaborator on this study and extensive care was taken to parallel the methodologies of the two studies to ensure comparability of findings. Second, although all breast cancer survivors were > 50 years of age, at baseline 6.5% of women in the breast cancer survivors cohort were not yet in menopause. This group also had higher education levels, earlier menarche, lower parity rates, lower personal fracture history, higher rates of calcium supplement use, and higher baseline 25OHD levels. During the design of our study, to avoid potential bias due to overmatching, we did not choose to match our controls based upon each of these factors and instead, took them into account in our regression analyses. As these characteristics would have been expected to be associated with lower risk of fracture, if anything, our findings would underestimate the risk of fracture among Chinese breast cancer survivors. Third, given the cross-sectional design of our study, our data do not allow for calculation of incidence rates of vertebral fracture over time, nor change in laboratory parameters. Our study was also not powered to look for subgroup analyses based upon treatment regimen, therefore we are unable to provide formal comparisons of fracture rates by type of treatment or duration of those treatments. Finally, our study population was limited to breast cancer survivors presenting for routine follow up at a major cancer hospital in Beijing, and therefore may not generalizable to all Chinese women with breast cancer, nor to other regions of China.

## Conclusion

In summary, our study found breast cancer survivors in China have a four-fold increased odds of prevalent vertebral fracture compared with age- and BMI-matched healthy women. However, rates of screening for osteoporosis and fracture risk by DXA were low reflecting a lack of standardization of care regarding screening, prevention and treatment of this problem. Future longitudinal studies are needed to elucidate the fracture risk specific to women who are premenopausal at the time of diagnosis and how risk relates to treatment regimens. Finally, infrastructure limitations such as lack of access to DXA imaging at cancer hospitals remain important barriers to timely intervention.

## Abbreviations

25OHD: 25-hydroxy vitamin D
AI: Aromatase inhibitors
BCS: Breast cancer survivors
BMI: Body mass index
CHCAMS: Cancer Hospital, Chinese Academy of Medical Sciences
CTIBL: Cancer treatment-induced bone loss
CTx: β-c-terminal telopeptide of type 1 collagen
DXA: Dual-energy x-ray absorptiometry
ER: Estrogen receptor
GnRH: Gonadotropin releasing hormone
HER2: Human epidermal growth factor receptor 2
ICD-9: International Conference for the Ninth Revision of the International Classification of Diseases
LMIC: Low- and middle-income countries
P1NP: Pro-collagen type 1 N propeptide
PK-VF: Peking Vertebral Fracture
PR: Progesterone receptor
SERM: Selective estrogen-receptor modulator
